# Parenting, mental health and economic pathways to prevention of violence against children in South Africa

**DOI:** 10.1016/j.socscimed.2020.113194

**Published:** 2020-07-21

**Authors:** L. Cluver, Y. Shenderovich, F. Meinck, M.N. Berezin, J. Doubt, C.L. Ward, J. Parra-Cardona, C. Lombard, J.M. Lachman, C. Wittesaele, I. Wessels, F. Gardner, J.I. Steinert

**Affiliations:** a Centre for Evidence-Based Intervention, Department of Social Policy & Intervention, University of Oxford, United Kingdom; b Department of Psychiatry and Mental Health, University of Cape Town, South Africa; c Institute of Criminology, University of Cambridge, United Kingdom; d Department of Applied Psychology, New York University, New York, USA; e Department of Psychology, University of Cape Town, South Africa; f Steve Hicks School of Social Work, The University of Texas at Austin, Texas, USA; g Biostatistics Unit, South African Medical Research Council, South Africa; h MRC/CSO Social and Public Health Sciences Unit, University of Glasglow, United Kingdom; i School of Social and Political Science, University of Edinburgh, United Kingdom; j Optentia, Faculty of Health Sciences, North-West University, South Africa; k TUM School of Governance, Technical University of Munich, Germany

**Keywords:** Violence, Parenting, Depression, Adolescence, Alcohol, Poverty

## Abstract

**Background:**

Parenting programs based on social learning theory have increasing empirical evidence for reducing violence against children. Trials are primarily from high-income countries and with young children. Globally, we know little about *how* parenting programs work to reduce violence, with no known studies in low or middle-income countries (LMICs). This study examines mechanisms of change of a non-commercialized parenting program, Parenting for Lifelong Health for Teens, designed with the World Health Organization and UNICEF. A cluster randomized trial showed main effects on parenting and other secondary outcomes. We conducted secondary analysis of trial data to investigate five potential mediators of reduced violence against children: improved parenting, adolescent behaviour, caregiver mental health, alcohol/drug avoidance, and family economic strengthening.

**Methods:**

The trial was implemented in rural South Africa with 40 sites, n = 552 family dyads (including adolescents aged 10–18 and primary caregivers). Intervention sites (n = 20) received the 14-session parenting program delivered by local community members, including modules on family budgeting and savings. Control sites (n = 20) received a brief informational workshop. Emotional and physical violence against children/adolescents and each potential mediator were reported by adolescents and caregivers at baseline and 9–13 months post-randomisation. Structural equation modelling was used to test simultaneous hypothesized pathways to violence reduction.

**Results:**

Improvements in four pathways mediated reduced violence against children: 1) improved parenting practices, 2) improved caregiver mental health (reduced depression), 3) increased caregiver alcohol/drug avoidance and 4) improved family economic welfare. Improved child behaviour was not a mediator, although it was associated with less violence.

**Conclusions:**

Simultaneously bolstering a set of family processes can reduce violence. Supporting self-care and positive coping for caregivers may be essential in challenging contexts. In countries with minimal or no economic safety nets, linking social learning parenting programs with economic strengthening skills may bring us closer to ending violence against children.

## 1. Introduction

Violence against children (VAC) – both physical and emotional – has severe detrimental impacts on children and adolescents (WHO, 2020). Long-term sequelae include increased mortality and morbidity, and impaired neurological function, mental health, education, and employment (Moffitt & Klaus-Grawe Think Tank, 2013; Teicher et al., 2016). Prevalence studies find elevated rates in low- and middle-income countries (LMIC), with Africa as the most affected region (Hillis et al., 2016).

Substantial evidence, primarily from high-income settings, suggests that parenting programs based on social learning theory may reduce violence against children (Barlow et al., 2006; Chen and Chan, 2016; Vlahovicova et al., 2017). Within LMIC there is a very new but emerging evidence-base, that also finds positive impacts of social learning-based parenting programs on parenting and reducing violence (Knerr et al., 2013; Parra-Cardona et al., 2018; Pedersen et al., 2019).

In developing effective parenting programs for LMIC, we need to understand mechanisms or mediators of violence reduction. To date, there are no known studies in either high- or low-income countries that quantitively investigate mediators from parenting programs to reduced violence against children. Two studies in Panama and Liberia with younger children (Giusto et al., 2017; Mejia et al., 2016) used qualitative methods to identify pathways of improved parenting behaviours, increased parental self-efficacy, and improved child behaviour. There is some evidence of mediators from parenting programs to improved child behaviour (rather than parental violence) in high-income countries (Forehand et al., 2014), and a very few in LMICs (Puffer et al., 2017). These find pathways of improved positive, involved and supervisory parenting, and reduced caregiver depression. (Shelleby et al., 2018).

Reviews of observational studies of child maltreatment suggest associated factors that may be modifiable by parenting programs: low parenting skills, caregiver depression or low self-esteem, caregiver stress, alcohol/drug use, and child behaviour problems (Meinck et al., 2014; Stith et al., 2009).

In addition, economic deprivation creates stressful environments that can reduce parenting capacity (Gershoff et al., 2007; Parra-Cardona et al., 2018; Meinck et al., 2014). Poverty, unemployment, and decreases in state benefit levels have all been associated with increased child maltreatment (e.g. Steinberg et al., 1981; Yang, 2015). A systematic review in 2014 found no parenting programs in LMIC that incorporated economic strengthening components (Marcus and Page, 2014). Since then, a small number of programs have combined parenting programmes with economic strengthening, all showing effectiveness in reducing parental violence towards children (Annan et al., 2013; Cluver et al., 2018; Ismayilova; Karimli, 2018).

No research has yet examined mechanisms of violence reduction for programs that combine parenting and economic strengthening. Although this trial only has two timepoints which test the outcome and possible mediators (baseline, followed by randomisation and then follow-up), our trial protocol specified that we would explore mediating pathways from other studies of child abuse prevention and the programme’s theory of change. Reviews identified parenting, caregiver mental health and child behaviour as possible mediators of violence reduction. During in-depth qualitative research led by UNICEF that took place alongside this trial (Doubt et al., 2018), program participants identified two additional pathways of change. The first was reduced stress on caregivers, leading to less use of alcohol and drugs as coping mechanisms. The second was reduced family conflict around money, due both to shared budget planning and higher collaboration around spending, and also due to improved income smoothing leading to reduced end-of-month severe hardship, and consequent lower stress.

This paper thus asks: Is reduced violence primarily driven by social learning mechanisms (i.e., improved parenting through better child behaviour, caregiver mental health, and alcohol/drug avoidance) or by alleviation of family distress linked to poverty, or a combination of both? This study tests these five hypothesized mediation pathways, within a cluster RCT of a parenting program with economic strengthening components, for families of adolescents in South Africa.

## 2. Methods

### 2.1. Intervention development

Parenting for Lifelong Health is an initiative co-developed by academics, the World Health Organisation (WHO), and UNICEF. Its goal is to develop and test parenting programs to prevent violence against children in LMIC. In South Africa, Parenting for Lifelong Health for Adolescents (locally named Sinovuyo Teen) was developed over five years in collaboration with a local NGO, Clowns Without Borders South Africa, and the National Department of Social Development. Stages of development and testing included qualitative piloting, adolescent advisory group participation, input from 50 academic and programming experts, and two pre-post trials with sequential refinement of the manual, followed by a full RCT (Cluver et al., 2016a, 2016b, 2016c). In brief, the RCT found significant reductions in physical and emotional abuse in caregiver report, whilst adolescents in both the intervention and control groups reported reductions in abuse. The intervention group reported improved parental supervision/monitoring and improved parental involved parenting, but not improved adolescent behaviour (adolescent and caregiver report). Caregivers in the intervention group reported improved caregiver mental health, reduced caregiver substance use and improved household economic welfare (adolescents did not report on these). All program materials are open access for non-profit use on the WHO and UNICEF websites http://www.who.int/violence_injury_prevention/violence/child/PLH-manuals/en/.

### 2.2. Study design and recruitment

A pragmatic cluster RCT including 552 families (adolescents aged 10–18 and primary caregivers) in 40 communities (32 rural villages and 8 peri-urban township clusters). The Eastern Cape – along with Limpopo – is South Africa’s poorest province (Statistics SA, 2016), with a remaining legacy of apartheid resulting in limited infrastructure and poor service delivery, and the area in which the study took place was identified by the national government and UNICEF South Africa as a priority for child abuse prevention. The research took place in collaboration with local traditional leaders and government. To reflect real-world service delivery, recruitment in April–August 2015 (prior to site randomisation) was informed by local chieftains, schools, community-selected representatives, and door-to-door visits, and was presented as general support for families in raising adolescent children. In order to prevent any risk of coercion, all referral sources were combined and many participants self-referred into the study after announcements in community meetings. Adolescents with physical or learning difficulties were included, unless these disabilities were so severe that they were unable to give informed consent or participate in any programme (n = 3). No other exclusion criteria applied, and we note that through this pragmatic RCT recruitment approach, the sample included those experiencing many other co-existing problems including severe abuse and other conditions such as mental health problems, HIV/AIDS, intimate partner violence, and substance use. Adolescents identified their primary caregiver as ‘the person who looks after you most’. Inclusion criteria were that caregivers and adolescents had to live in the same dwelling for at least four nights per week, with no requirement for a biological relationship.

Original power calculations for the trial did not include mediation models, and therefore power calculations for these were not conducted post-hoc (Dziak et al., 2018; Gelman, 2019). The trial was powered to 80% and 95% confidence with 40 clusters for a minimum detectable effect size of 0.36 for the main outcome of violence against children. Power calculations were based on a two-level multi-level model. The 0.80 refers to the desired level of power and alpha 0.05 to the level of statistical significance for the ability to detect the overall treatment effect. We present all relevant parameters for a power calculation in supplementary material for future studies that may wish to plan similar analyses (MacKinnon et al., 2007).

Randomisation was stratified by rural/urban location and conducted after baseline using a random number generator by an independent, blinded statistician (CL). Complete randomisation within strata used a ratio of 1:1 intervention: control. The sample included 270 families in the intervention arm and 282 families in the control arm (mean 14 families per cluster, SD 1.9). Blinding of participants and program providers is not feasible for parenting programs.

Ethical approval was given by the University of Oxford (SSD/CUREC2/11–40), University of Cape Town (PSY2014–001), and government Departments of Social Development and Education. The protocol was published (Cluver et al., 2016) and the trial registered on the Pan-African Clinical Trials Registry PACTR201507001119966 on 27/4/2015, but did not include the exploratory mediation analyses conducted in this paper. An independent trial steering committee oversaw trial conduct. Written informed consent was given by all adults and adolescents. No monetary incentives were given for participation, but all families were given snacks at baseline and small food parcels at follow-up as thanks. Confidentiality was maintained unless participants were at risk of significant harm or asked for support. In cases of severe abuse, rape, suicide attempts or other significant harm, 33 immediate referrals and follow-up assistance were made to social and health services.

### 2.3. Procedures

Self-report tablet-based questionnaires were completed by primary caregivers and adolescents at baseline and 9–13 months (mean 12 months) post-randomisation. A very brief questionnaire including only primary outcomes was completed at one-month post-program, and therefore was not used in this analysis. Data collectors supported the process and audio-computer assisted self-interviewing was modified after pre-piloting for low literacy levels. Participants chose their language (isiXhosa or English) and privacy was ensured. There was a deviation from the protocol due to extended political violence related to the 2016 elections, with riots, road blockages and petrol-bombing that substantially affected delivery and data collection. This resulted in a shift of the planned final data collection stage from 19 months post-program to 9–13 months post randomisation.

Intervention clusters received the Parenting for Lifelong Health for Teens program comprising 14 weekly sessions, attended by adolescents and their primary caregivers. The program was designed for low-resource settings with no technology (such as video) or literacy requirements, and used non-didactic, collaborative learning processes including role-plays, activities, discussions, and songs. Sessions lasted 1.5–2 h and included praise, managing anger and stress, collaborative problem-solving, planning together to protect adolescents from community violence, and two sessions focused on family budgeting, saving and financial planning for the future. The program was delivered by local community members, who were trained by a local NGO, Clowns Without Borders South Africa, and supported through weekly supervision. Sessions were delivered in locally available spaces, such as community halls. Control clusters received a community level one-day hygiene promotion workshop (5 h), also delivered by Clowns Without Borders South Africa, which focused on handwashing skills for children, delivered through performance and activities. All children received a soap which – when used – had a small toy inside. The choice of active placebo was made by the local communities, who were concerned about child sanitation after a period of drought.

### 2.4. Measures

All outcomes and potential mediators were measured for the past month, and are available online https://www.unicef-irc.org/research-family-and-parenting/. Measures were reported independently by both adolescents and primary caregivers at baseline and 9–13 month follow-up. Child and caregiver reports were combined (summed into a total aggregate score) in order to explore family-level processes, incorporate both adult and adolescent perspectives, and to reduce the impact of social desirability bias in reporting physical abuse, emotional abuse, parental supervision, involved parenting, and child problem behaviour. For caregiver depression, caregiver alcohol/drug use, and household economic welfare only caregiver report was measured: in rural contexts it is often considered inappropriate to inform children about household finances, and two thirds of adolescents in this sample reported no insight into the family’s financial management.

### 2.5. Outcome

Physical and emotional violence at baseline and follow-up were measured using adolescent and caregiver-report on 14 items of the relevant subscales of the International Society for Prevention of Child Abuse and Neglect Screening Tool for Trials (ICAST-Trial) (Meinck et al., 2018). *Potential mediators:* Mediators were measured at baseline and follow-up, and analyses controlled for baseline values of all mediator and outcome variables. Parenting comprised child and caregiver-report on 20 items of the parental supervision and positive involved parenting subscales of the Alabama Parenting Questionnaire (Frick, 1991). Caregiver depression used seven items from the Centre for Epidemiologic Studies Depression Scale (Radloff, 1977). Caregiver alcohol and drug use used an adapted version of the WHO Alcohol Use Disorders Identification Test (3 items) and was coded ordinally as no, regular, and very frequent alcohol or drug consumption (Saunders et al., 1993). Child and caregiver-report of child problem behaviour used the 35-item Child Behavior Checklist rule-breaking and aggression sub-scales (Achenbach, 2000). For each of the above scales, individual items were aggregated into a total sum score, similar to the approach used in previous psychological literature. Household economic welfare used a four-item scale measuring monthly consistent access to necessities including food, electricity, communication (airtime), and transport (Morduch, 1995; Townsend, 1995), which was aggregated into a principal-component weighted scale centered around 0, thus following conventions in the poverty measurement literature (Filmer and Pritchett, 2001; Sahn and Stifel, 2003).

### 2.6. Covariates

All analyses controlled for baseline values of violence and of all hypothesized mediators to account for potential differences between treatment and control group at the study’s outset. Additional covariates included child and caregiver gender, child and caregiver age, rural/urban location, whether the caregiver was a biological parent of the adolescent, and household living standards using an asset index of the eight most highly-voted necessities for households with children, identified through the South African National Social Attitudes Survey (Wright, 2008) and scored based on principal-component-weighting.

### 2.7. Analyses

Analyses used intention-to-treat (ITT) for all clusters and families irrespective of intervention uptake, and included families who were no longer living together at follow-up (n = 53). The full sample was used in order to allow testing of intervention impact on hypothesized mediators. Potential mediators were first tested separately following methods to estimate average causal mediation effects (ACME) (Imai et al., 2010; Imai et al., 2011). Using nonparametric bootstrapping procedure with 1000 resamples, this analysis splits the average treatment effect (ATE) of the Sinovuyo Teen programme both into an *indirect effect* (the ACME) that runs through an observed intermediate variable and also into a *direct effect* that runs through unobserved channels. Mediation analyses controlled for baseline values, and standard errors were clustered at the village level, i.e., the unit of randomisation. In a final step, using the full sample, all hypothesized mediating variables (Hayes, 2009) were entered simultaneously into a linear structural equation model (parametric estimation), and analyses controlled for baseline measures of all mediators and outcome. Interactions were not tested for as we did not have time separation between the mediators and outcome. Goodness of fit for the final model (without clustering) was assessed using the Comparative Fit Index (CFI), the Root Mean Standard Error of Approximation (RMSEA), and the Standardized Root Mean Square Residual (SRMR). We also report χ^2^ fit statistics but acknowledge that the test is inflated by sample size. Following Brown (2015), the model was refined for improved goodness of fit by taking modification indices into account and correlating respective item residuals.

## 3. Results

Basic sociodemographic characteristics of the study sample are reported in Cluver et al. (2018). Baseline and follow-up values of the outcome and mediating variables are reported in Table 1. Internal consistency of all aggregated variables was high and ranged between alpha values of 0.71 and 0.89. Attrition was low, with 11% of the sample missing items or one of the caregiver/adolescent responses, and missing status was similar across arms and not associated with the intervention. Given these, complete case analysis was conducted within complete dyads.

### 3.1. Mediation

Primary analyses are reported in Cluver et al., 2018. In summary, caregivers and adolescents in the intervention group reported reduced physical and emotional violence, improved parental monitoring, and involved parenting (all based on combined child- and caregiver-report). Caregivers in the intervention group reported improved mental health, alcohol/drug avoidance, and household economic welfare (based on caregiver report only). Control group adolescents also reported reduced violence, and there were no differences for child behaviour problems.

Table 2 shows each mediator tested separately. In column (1), we show parametric estimates of the program’s effect on each respective mediator. In columns (2)–(4), we show nonparametric (based on bootstrapping with 1000 simulations) estimates of the ACME, direct, and total effect. The ACME is the statistic of interest and indicates a) whether a mediation effect exists and b) in which direction it runs. Three of the four tested pathways were significant: improved parenting with an average causal mediation effect (ACME) of −2.08 (95% CI [−3.08, −1.29]), improved caregiver mental health ACME −1.10 (95% CI [ −2.00, −0.47]), improved caregiver alcohol/drug avoidance ACME – 0.44 (95% CI [ −0.90, −0.10]), and improved economic welfare ACME −0.39 (95% CI [ −0.75, −0.14]). Improved adolescent behaviour was not a mediator.

**Fig. 1.**
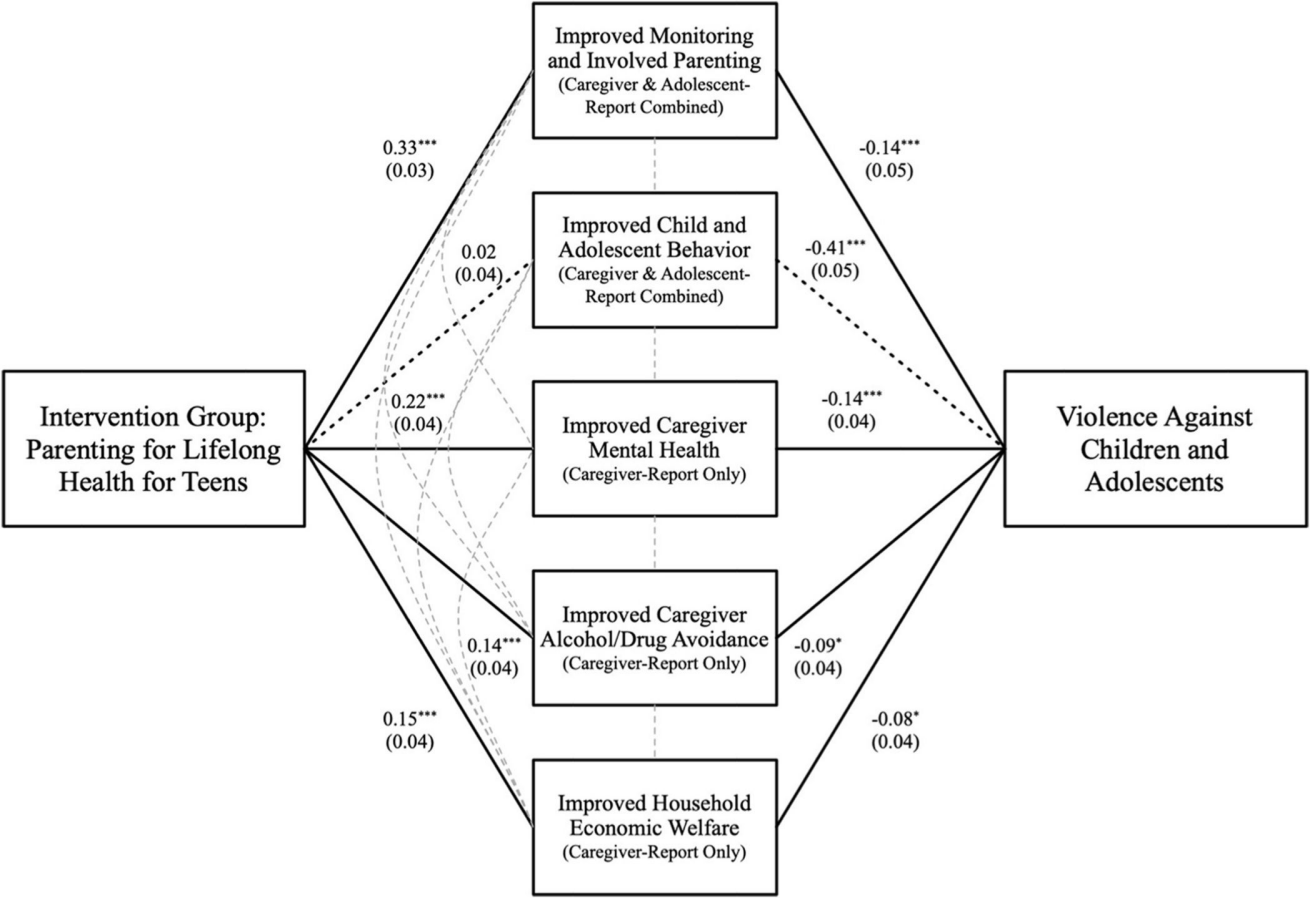
Structural Equation Model of Program Mediators, Notes: ***p < 0.001, **p < 0.01, *p < 0.05. All coefficients shown are standardized.

In a subsequent step, all four mediators were entered simultaneously into a structural equation model. The final structural model (see Fig. 1) showed moderate to good fit with CFI = 0.981, RMSEA = 0.080, SRMR = 0.011 and χ^2^ = 20.491***. Effects on violence reduction ran through four indirect pathways: improvements in parenting skills, caregiver mental health, alcohol/drug avoidance, and family economic welfare. At follow-up, intervention clusters had improved parenting (*β* = 0.33, SE = 0.033, p < 0.001), improved mental health (less depression) (*β* = 0.22, SE = 0.042, p < 0.001), improved alcohol and drug avoidance (*β* = 0.14, SE 0.040, p < 0.001), and increased likelihood of reaching the end of the month with enough food, electricity, and transport (β = 0.15, SE = 0.042, p < 0.001). There was no pathway from the intervention to adolescent behaviours (β = 0.02, SE = 0.039, p = 0.616). All mediating variables were directly associated with reductions in violence against children: improved parenting (β = −0.14, SE = 0.050, p = 0.005), improved mental health (β = −0.14, SE = 0.041, p < 0.001), improved alcohol/drug avoidance (β = −0.09, SE = 043, p = 0.029) and economic welfare (β = −0.08, SE = 0.041, p = 0.048). Improved adolescent behaviour (whilst not a significant mediator), was associated with reduced violence (β = 0.41, SE = 0.046, p < 0.001).

## 4. Discussion

This study is, to our knowledge, the first in a low or middle income country to quantitatively examine mediators of a parenting program on reduction of violence against children (WHO, 2016). Results indicate four pathways: improved parenting skills, caregiver mental health, alcohol/drug avoidance, and household-level economic welfare. Findings suggest the continuing importance of supporting caregivers with strategies for protective parenting. They also suggest that improving caregiver wellbeing and coping strategies leads to improvements for children. Finally, they suggest that combining parenting and economic strengthening programs – particularly in poor communities – may boost effectiveness against violence. These implications are supported by qualitative work from other low-resource settings, for example, caregivers in Latin America and Africa describing overwhelming parenting stress related to poverty (Doubt et al., 2018; Lachman et al., 2016; Parra-Cardona et al., 2009).

This study has a number of limitations. First, our measures were limited to baseline, randomisation into trial arms (after baseline) and follow-up (mean 12 months after randomisation). Thus, hypothesized mediators and the violence reduction outcome were measured at the same time, although both were able to measure change by controlling for baseline scores within the model. Although mediation analyses with two time points are common in the literature (Forehand et al., 2014; Gardner et al., 2010), future studies could valuably use multiple follow-up time points. Second, there are always limitations of mediation analyses in establishing causality of discrete intervention components: improvements in economic welfare mediated reductions in violence, but these improvements may have been due to other aspects of the intervention in addition to the economic sessions – for example, reduced caregiver alcohol/drug use may have allowed more money to be allocated to food. Approaches such as factorial experiments could be used in future studies to isolate core components.

Third, the current analysis weighted child and caregiver responses equally, due to limited statistical power and linked to this, a limited number of degrees of freedom. Future analyses could additionally model variables such as parenting and violence as latent factors. Fourth, due to the real-world sample, the violence outcome was skewed. However, these complex mediation models were impossible to compute with a non-linear link and transformation does not solve high numbers of zero scores. Therefore, we used MPlus which has the capacity to conduct SEM on skewed distributions and checked goodness of fit indicators which were good. Fifth, the trial was conducted by the program developers, and additional studies should be conducted independently. Sixth, due to sustained election violence, the study was not able to conduct follow-up beyond 9 months. A recent review of parenting in conflict zones finds that impacts on children (such as behaviour changes) may be delayed and therefore longer follow-up times are needed (Murphy et al., 2017). Seventh, the trial was only powered to detect substantially larger than average effects for parenting programs on violence, thus likely under-estimated program impacts on both mediators and outcome. Eighth, parenting was measured through adolescent and caregiver report. Whilst home observations have strong external validity for younger children, it is difficult to get reliable observations of adolescent-caregiver interactions with an interviewer present, and in rural areas where large families live in one room households. Globally, studies find discrepancies between adolescent and caregiver report (or perception) of both parenting and adolescent behaviours, and so we combined and summed both reports to gain a family-level average.

Strengths of the study include the pragmatic randomized trial method, which uses real-world recruitment and service delivery platforms to provide high external validity. In particular, sampling purposefully reflected real-world service delivery in African contexts, with participants referred by a range of community members, state services, traditional leaders and self-referrals, and no exclusion criteria. In contexts of very weak social and police services (as across Africa) community approaches are the most feasible to reach families experiencing risk factors for violence. However, we note that our study area included rural areas and peri-urban townships, but no major inner-city areas – which may be characterised by differing family challenges. It is important to recognise the additional needs of families with severe disabilities, and the research team are currently working with UNICEF’s disability team and the Special Olympics to adapt and deliver parenting support. We also note that the study setting in South Africa has implications for the economic aspect of the intervention: most families had access to small, regular state poverty alleviation grants of around $30/month for the household. In contexts where families may have no income at all, budgeting and savings plans may require adaptation.

The trial shows that violence reduction through simultaneous behavioural and economic strengthening pathways is possible during political unrest and in a very low-resource area. We used intention-to-treat analyses and standardized outcome measures, including actual violence, rather than commonly-used proxies such as parenting stress or views about violence, and combined caregiver and child report wherever feasible.

These findings may also reflect some of those seen in other fields of violence prevention. Studies of intimate partner violence in South Africa, Cote D’Ivoire and Tanzania have shown positive impacts of programs combining economic strengthening (such as microfinance or village savings and loans groups) with gender norms training (Gupta et al., 2013; Harvey et al., 2018; Pronyk et al., 2006). Recent global initiatives to prevent violence against children highlight the importance of both parenting programs and economic strengthening (Know Violence in Childhood, 2017; World Health Organisation, 2016). This reflects a broader conceptual shift led by the Sustainable Development Goals, which conceptualise goals such as poverty reduction, mental health and violence prevention as interlinked and inter-dependent.

## 5. Conclusion

These findings can inform the scale-up of evidence-based parenting programs for low-resource settings (Puffer et al., 2017). The Parenting for Lifelong Health for Teens program is now used by a range of NGOs, governments and donors, including USAID-PEPFAR, UNICEF, Catholic Relief Services and Pact, and included in the WHO ‘INSPIRE’ policy guidelines. It is currently implemented in 15 low- and middle-income countries, reaching an estimated 600,000 families by 2021.

Further important implementation science questions remain. These include how to most cost-effectively combine strategies within programs, avoiding excessively long or complex delivery processes, training and supporting local staff in sustainable program implementation (Ward et al., 2015). Nonetheless, there is growing advocacy in both high- and low-income settings for combining parenting support, mental health and economic strengthening (Richter and Naiker, 2015; WHO, 2020). This study provides empirical support for doing so. It indicates a next step in the field of parenting programs: capitalizing on the strong evidence-base for social learning methods, and building on this with approaches from other fields such as development economics. Through such linkages, we can reduce violence against children in the world’s highest-risk, lowest-resource contexts.

## Supplementary Material

Supp Material

## Figures and Tables

**Table 1:** Sample characteristics.

	Baseline		Follow-Up	

Mean (SD)	Treatment	Control	Treatment	Control
Outcome Variable (1) Emotional and Physical Violence against Children* (Cronbach’s α = 0.81)	15.69 (16.11)	14.14 (14.55)	7.28 (10.85)	8.27 (10.05)
Mediating Variables (2) Monitored and Involved Parenting* (Cronbach’s α = 0.79)	9.61 (18.59)	11.83 (18.47)	29.00 (18.08)	15.64 (18.99)
(3) Child Problem Behaviour*(Cronbach’s α = 0.89)	33.18 (16.56)	31.98 (15.66)	24.44 (14.44)	24.15 (14.75)
(4) Caregiver Depression (Cronbach’s α = 0.71)	23.13 (11.78)	24.90 (12.09)	11.30 (9.78)	16.82 (11.13)
(5) Caregiver Alcohol/Drug Use	0.25 (0.50)	0.30 (0.54)	0.16 (0.40)	0.33 (0.62)
(6) Household Economic Welfare (Cronbach’s α = 0.72)	0.04 (1.68)	−0.04 (1.64)	0.29 (1.60)	−0.28 (1.49)
N	270	282	264	278

Notes:

* based on combined (summed) caregiver and child reports. Variables (1)–(4) are continuous scale scores, Variable (5) here reported as binary, coded 1 if any reported drug use or more than three drinks per one day in the past month. Variable (6) is a continuous scale score based on principal component weighting of access to four necessities (e.g., transport, food, electricity, communication), centered around 0. Cronbach’s alpha reported for the baseline scale.

**Table 2:** Mediation Analysis for the Outcome of violence against children (ICAST).

Potential Mediator	Effect of Program Participation on Mediator (1)	Average Causal Mediation Effect (2)	Direct Effect (3)	Total Effect (4)	Sample Size (5)
Improved Monitoring and Involved Parenting	12.87*** [9.87, 15.88]	−2.08 [−3.08, −1.29]	1.21 [−0,62, 3.20]	−0.89 [−2.89, 1.09]	489
Improved Adolescent Behaviour	−0.44 [−2.94, 2.06]	0.16 [−0.75, 1.06]	−1.01 [−2.41, 0.51]	−0.86 [−2.66, 0.97]	489
Improved Caregiver Mental Health (Reduced Depression)	5.16*** [2.96, 7.36]	−1.10 [ −2.00, −0.47]	0.27 [−1.39, 2.08]	−0.84 [−2.62, 0.99]	489
Improved Caregiver Alcohol/Drug Avoidance	0.15*** [0.06, 0.24]	−0.44[−0.90, −0.10]	−0.52 [−2.08, 1.16]	−0.91 [−2.62, −0.90]	489
Improved Household Economic Welfare	0.49*** [0.21, 0.76]	−0.39[ −0.75, −0.14]	−0.52 [−2.23, 1.33]	−0.93 [−2.85, 1.04]	489

Notes:

* p < 0.05,

** p < 0.01,

*** p < 0.001, 95% CIs in square brackets. Coefficients are unstandardized and standard errors were clustered at the village level. Column (1) represents the parametric inference of the program’s effect on the respective mediator. Estimates shown in columns (2)–(4) are based on a nonparametric bootstrap procedure with 1000 simulations (consequently significance levels are not provided and CI range is used). Each mediator was tested individually. All analyses control for caregiver and adolescent age and sex, rural/urban location, biological/non-biological relationship, and household living standards. Sample size varies for tested mediators as combined adolescent and caregiver scores could only be calculated if both pair members were interviewed at follow-up. As each mediation model was run separately and the direct and indirect effect varies by mediator, the total effect on violence differs by model – varying from −0.84 to −0.93.
