# Supplementary material for: Parenting, mental health and economic pathways to prevention of violence against children in South Africa
Source: Soc Sci Med. Author manuscript; Available in PMC 2024 Jan 11. (PMC10782842; doi:10.1016/j.socscimed.2020.113194)
Supplement: Supp Material [file NIHMS1907918-supplement-Supp_Material.docx]

**Appendix**

Table 1. Correlations between Caregiver and Adolescent Report

| **Construct** | **Correlation Caregiver and Adolescent Report** |
| --- | --- |
| Teen Problem Behaviour (baseline) | 0.24 |
| Teen Problem Behaviour (endline) | 0.27 |
| Monitored and Involved Parenting (baseline) | 0.11 |
| Monitored and Involved Parenting (baseline) | 0.07 |

Table 2. Results for the Final Structural Equation Model *with* Clustered Standard Errors

| **Structural Model** | | | | | | |
| --- | --- | --- | --- | --- | --- | --- |
|  | **Violence Against Children and Adolescents** | **Improved Monitored and Involved Parenting** | **Improved Adolescent Problem Behaviour** | **Improved Caregiver Mental Health (Reduced Depression)** | **Improved Caregiver Substance Use (Reduced Substance Use)** | **Improved Household Economic Welfare** |
| Trial Arm | 0.05  [-0.02, 0.12] | 0.33***  [0.26, 0.40] | -0.02  [-0.10, 0.06] | 0.22***  [0.13, 0.31] | 0.14***  [0.06, 0.22] | 0.15***  [0.07, 0.23] |
| Improved Monitoring and Involved Parenting | -0.14***  [-0.25, -0.04] | - |  |  |  |  |
| Improved Adolescent Problem Behaviour | -0.41***  [-0.52, -0.29] |  | - |  |  |  |
| Improved Caregiver Mental Health (Reduced Depression) | -0.14***  [-0.23, -0.05] |  |  | - |  |  |
| Improved Caregiver Substance Use (Reduced Substance Use) | -0.09  [-0.21, 0.02] |  |  |  | - |  |
| Improved Household Economic Welfare | -0.08**  [-0.14, -0.02] |  |  |  |  | - |
|  |  |  |  |  |  |  |
| Rural | 0.05  [-0.04, 0.14] |  |  |  |  |  |
| Household Asset Index | -0.09**  [-0.16, -0.02] |  |  |  |  |  |
| Female (Adolescent) | 0.08  [-0.01, 0.16] |  |  |  |  |  |
| Female (Caregiver) | 0.12***  [0.05, 0.18] |  |  |  |  |  |
| Age (Adolescent) | -0.06  [-0.16, 0.04] |  |  |  |  |  |
| Age (Caregiver) | -0.06  [-0.13, 0.00] |  |  |  |  |  |
| Biological Parent | 0.03  [-0.04, 0.10] |  |  |  |  |  |
| Baseline Monitoring and Involved Parenting | 0.02  [-0.09, 0.13] |  |  |  |  |  |
| Baseline Adolescent Problem Behaviour | 0.15***  [0.06, 0.25] |  |  |  |  |  |
| Baseline Caregiver Mental Health | -0.04  [-0.12, 0.03] |  |  |  |  |  |
| Baseline Caregiver Substance Use | 0.03  [-0.05 0.11] |  |  |  |  |  |
| Baseline Household Economic Welfare | 0.03  [-0.06, 0.11] |  |  |  |  |  |
| Baseline Violence Against Children and Adolescents | 0.11  [-0.03, 0.26] |  |  |  |  |  |

| **Correlated Item Residuals** |  |
| --- | --- |
| Improved Caregiver Mental Health (Reduced Depression)  X Improved Monitoring and Involved Parenting | 0.13***  [0.04, 0.23] |
| Improved Caregiver Mental Health (Reduced Depression)  X Improved Adolescent Problem Behaviour | 0.13***  [0.03, 0.25] |
| Improved Caregiver Mental Health (Reduced Depression)  X Improved Household Economic Welfare | 0.13***  [0.04, 0.21] |
| Improved Caregiver Mental Health (Reduced Depression)  X Improved Caregiver Substance Use (Reduced Substance Use) | 0.10**  [0.03, 0.18] |
| Improved Monitoring and Involved Parenting  X Improved Adolescent Problem Behaviour | 0.31***  [0.22, 0.40] |
| Improved Monitoring and Involved Parenting  X Improved Household Economic Welfare | -0.06  [-0.16, 0.04] |
| Improved Monitoring and Involved Parenting  X Improved Caregiver Substance Use (Reduced Substance Use) | 0.05  [-0.02, 0.12] |
| Improved Adolescent Problem Behaviour X Improved Household Economic Welfare | 0.00  [-0.09, 0.09] |
| Improved Adolescent Problem Behaviour X Improved Caregiver Substance Use (Reduced Substance Use) | 0.01  [-0.08, 0.09] |
| Improved Household Economic Welfare X Improved Caregiver Substance Use (Reduced Substance Use) | -0.01  [-0.09, 0.07] |
| **Model Fit** | |
| **BIC** | 44732.69 |
| **AIC** | 44569.19 |
| **Observations = 489** |  |

*Notes: *p<0.1, **p<0.05, ***p<0.01. Standardised estimates shown, 95% confidence intervals in parentheses. Coefficients are based on a DWLS estimator. The table shows results with the best model fit*

Table 3. Results for the Final Structural Equation Model *without* Clustered Standard Errors

| **Structural Model** | | | | | | | |
| --- | --- | --- | --- | --- | --- | --- | --- |
|  | **Violence Against Children and Adolescents** | **Improved Monitored and Involved Parenting** | **Improved Adolescent Problem Behaviour** | | **Improved Caregiver Mental Health (Reduced Depression)** | **Improved Caregiver Substance Use (Reduced Substance Use)** | **Improved Household Economic Welfare** |
| Trial Arm | 0.05  [-0.03, 0.14] | 0.33***  [0.26, 0.40] | 0.02  [-0.06, 0.10] | | 0.22***  [0.14, 0.30] | 0.14***  [0.06, 0.22] | 0.15***  [0.07, 0.23] |
| Improved Monitoring and Involved Parenting | -0.14***  [-0.24, -0.04] | - |  | |  |  |  |
| Improved Adolescent Problem Behaviour | 0.41***  [0.32, 0.50] |  | - | |  |  |  |
| Improved Caregiver Mental Health (Reduced Depression) | -0.14***  [-0.22, -0.06] |  |  | | - |  |  |
| Improved Caregiver Substance Use (Reduced Substance Use) | -0.09**  [-0.18, -0.01] |  |  | |  | - |  |
| Improved Household Economic Welfare | -0.08**  [-0.16, 0.00] |  |  | |  |  | - |
|  |  |  |  | |  |  |  |
| Rural | 0.05  [-0.03, 0.13] |  |  | |  |  |  |
| Household Asset Index | -0.09**  [-0.18, -0.01] |  |  | |  |  |  |
| Female (Adolescent) | 0.08*  [0.00, 0.15] |  |  | |  |  |  |
| Female (Caregiver) | 0.12***  [0.04, 0.19] |  |  | |  |  |  |
| Age (Adolescent) | -0.06  [-0.15, 0.03] |  |  | |  |  |  |
| Age (Caregiver) | -0.06  [-0.15, 0.02] |  |  | |  |  |  |
| Biological Parent | 0.03  [-0.05, 0.12] |  |  | |  |  |  |
|  |  |  |  | |  |  |  |
| Baseline Monitoring and Involved Parenting | 0.02  [-0.08, 0.12] |  |  | |  |  |  |
| Baseline Adolescent Problem Behaviour | -0.15***  [-0.25, -0.05] |  |  | |  |  |  |
| Baseline Caregiver Mental Health | -0.04  [-0.12, 0.04] |  |  | |  |  |  |
| Baseline Caregiver Substance Use | 0.03  [-0.05, 0.11] |  |  | |  |  |  |
| Baseline Household Economic Welfare | 0.03  [-0.06, 0.11] |  |  | |  |  |  |
| Baseline Violence Against Children and Adolescents | 0.11***  [0.03, 0.20] |  |  | |  |  |  |
| **Correlated Item Residuals** | | | |  | | | |
| Improved Caregiver Mental Health (Reduced Depression)  X Improved Monitoring and Involved Parenting | | | | 0.13**  [0.05, 0.22] | | | |
| Improved Caregiver Mental Health (Reduced Depression)  X Improved Adolescent Problem Behaviour | | | | 0.14**  [-0.23, -0.05] | | | |
| Improved Caregiver Mental Health (Reduced Depression)  X Improved Household Economic Welfare | | | | 0.13***  [0.04, 0.21] | | | |
| Improved Caregiver Mental Health (Reduced Depression)  X Improved Caregiver Substance Use (Reduced Substance Use) | | | | 0.10**  [0.02, 0.19] | | | |
| Improved Monitoring and Involved Parenting  X Improved Adolescent Problem Behaviour | | | | 0.31***  [-0.39, -0.23] | | | |
| Improved Monitoring and Involved Parenting  X Improved Household Economic Welfare | | | | -0.06  [-0.15, 0.03] | | | |
| Improved Monitoring and Involved Parenting  X Improved Caregiver Substance Use (Reduced Substance Use) | | | | 0.05  [-0.04, 0.14] | | | |
| Improved Adolescent Problem Behaviour  X Improved Household Economic Welfare | | | | 0.00  [-0.09, 0.09] | | | |
| Improved Adolescent Problem Behaviour  X Improved Caregiver Substance Use (Reduced Substance Use) | | | | -0.01  [-0.09, 0.08] | | | |
| Improved Household Economic Welfare  X Improved Caregiver Substance Use (Reduced Substance Use) | | | | -0.01  [-0.10, 0.08] | | | |
| **Model Fit** | | | | | | | |
| **χ^2^** | | | | 20.491*** | | | |
| **CFI** | | | | 0.981 | | | |
| **RMSEA** | | | | 0.080 | | | |
| **SRMR** | | | | 0.011 | | | |
| **BIC** | | | | 44992.77 | | | |
| **AIC** | | | | 44653.19 | | | |
| **Observations = 489** | | | |  | | | |

*Notes: *p<0.1, **p<0.05, ***p<0.01. Standardised estimates shown, 95% confidence intervals in parentheses. Coefficients are based on a DWLS estimator. The table shows results with the best model fit*

Table 4. Intra-Cluster Correlations (ICCs) of Mediator and Outcome Variables at 9-13-month Follow-up

| **Measures** | **ICC by study cluster** | **N observations** |
| --- | --- | --- |
| Violence against children | 0.02 | 542 |
| Monitoring and Involved Parenting | 0.18 | 494 |
| Adolescent Behaviour | 0.08 | 494 |
| Caregiver Mental Health (Depression) | 0.08 | 540 |
| Caregiver Alcohol/Drug Avoidance | 0.07 | 540 |
| Household Economic Welfare | 0.04 | 540 |
